# Innovative wearable technology for continuous echocardiographic monitoring: first-in-neonate case report

**DOI:** 10.3389/fped.2025.1567386

**Published:** 2025-04-15

**Authors:** Yan Wang, Gangjun Wu, Jianqiang Song, Haibo Song, Qinjun Chu

**Affiliations:** ^1^ Department of Anesthesiology and Perioperative Medicine, Zhengzhou Center Hospital Affiliated to Zhengzhou University, Zhengzhou, Henan, China; ^2^ Research and Development, Sichuan Wismed Medical Technology, Chengdu, Sichuan, China; ^3^ Department of Anesthesiology, West China Hospital of Sichuan University, Chengdu, Sichuan, China

**Keywords:** wearable ultrasound, continuous echocardiographic monitoring, neonate, gastrointestinal bleeding, case report

## Abstract

Neonates in the early stage are in the transition from fetal to adult circulation, accompanied by complex hemodynamic changes, which are prone to aggravate the condition of the circulatory system during the perioperative period. Here, we present a case of a 3-day-old neonate with upper gastrointestinal bleeding caused by multiple esophageal and gastric ulcers. To achieve accurate circulation management, we utilized a novel wearable ultrasound device for non—invasive, continuous echocardiographic monitoring throughout general anesthesia gastroscopy and postoperative NICU care. The case demonstrates the feasibility of continuous echocardiographic monitoring in neonates and offers new ideas for circulatory monitoring and management in critical neonates.

## Introduction

Neonates and infants are the group of patients at highest risk for perioperative anesthetic complications, and hemodynamic changes are associated with an increased risk of death (1). Echocardiography can easily and rapidly quantify ventricular function and size to guide circulation management (2), which is widely used in the cardiac evaluation of critically ill neonates. This case introduces the application of a new type of wearable echocardiography monitoring in the perioperative period of neonates, which provides a basis for circulation management through real-time and continuous echocardiographic views.

## Case report

A neonate, 51 cm in height and 3.75 kg in weight, who had just been delivered by cesarean section, was admitted to the neonatal intensive care unit (NICU) due to blood glucose as low as 1.3 mmol/L. Following admission, the neonate exhibited symptoms of gastrointestinal hemorrhage, presenting with coffee ground emesis and red mucus. Treatment included fasting, gastrointestinal decompression, intragastric hemostatic medication, and a transfusion of 40 ml of cryoprecipitate to address the hemorrhage. But following 3 days of symptomatic treatment, the neonate continued to vomit coffee grounds and had bloody substance drawn from the gastric tube. A painless gastroscopy was recommended to determine the source of bleeding.

**Figure 1:**
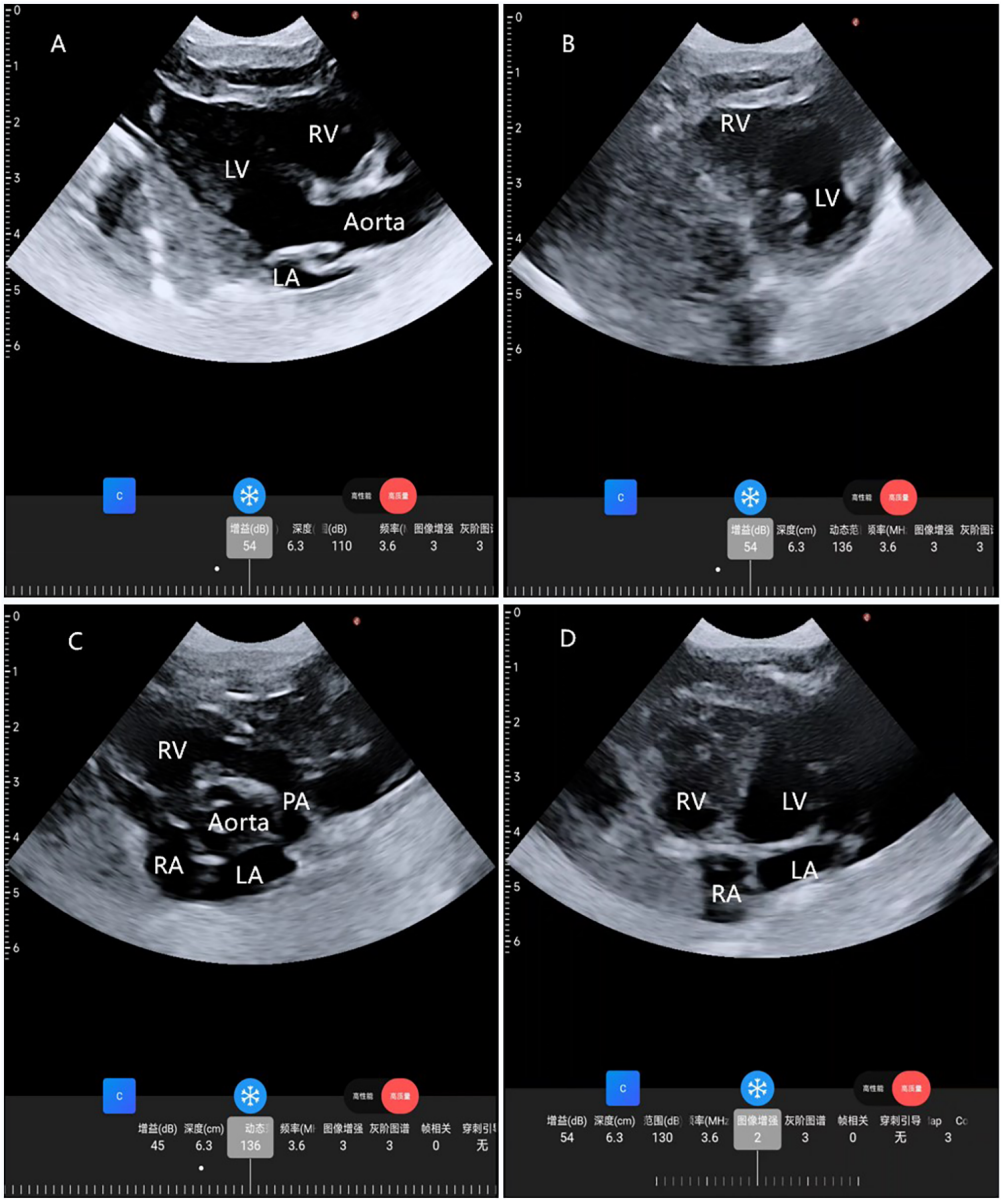
Cardiac images from four standard views taken by a wearable cardiac ultrasound monitoring imager. **(A)** Parasternal long-axis view; **(B)** parasternal LV short-axis view; **(C)** parasternal aortic short-axis view; **(D)** apical 4-chamber view. RA, right atrium; RV, right ventricle; LA, left atrium; LV, left ventricle; PA, pulmonary artery.

**Figure 2:**
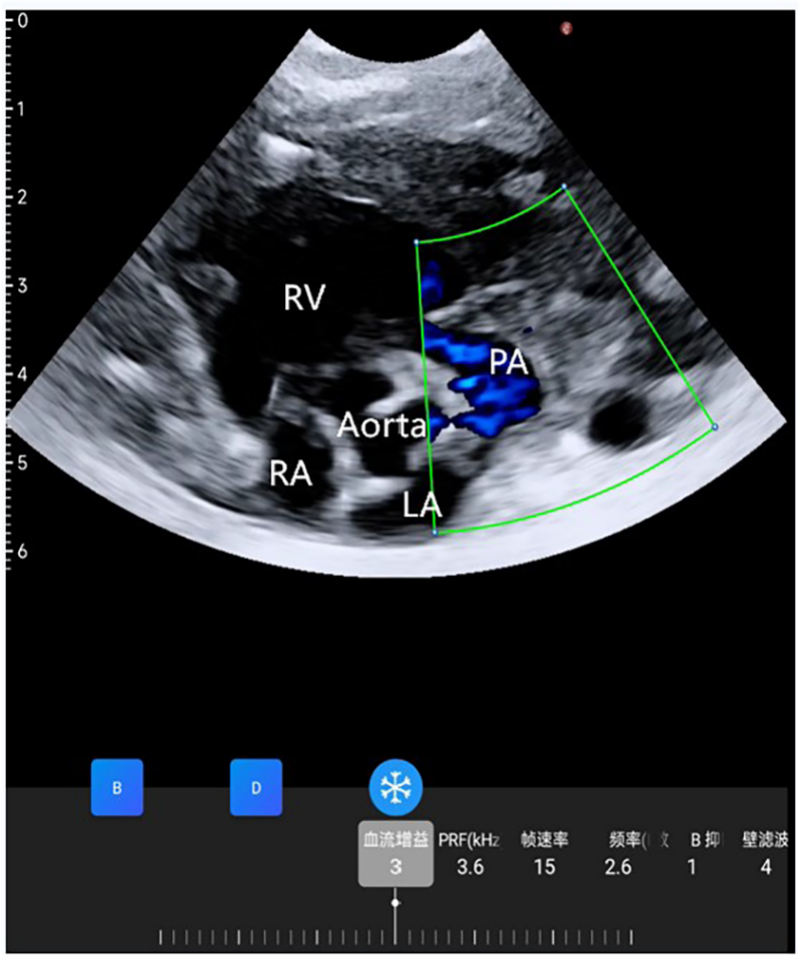
Wearable ultrasonic colour flow doppler examination between the aorta and pulmonary artery did not find a PDA.

**Figure 3:**
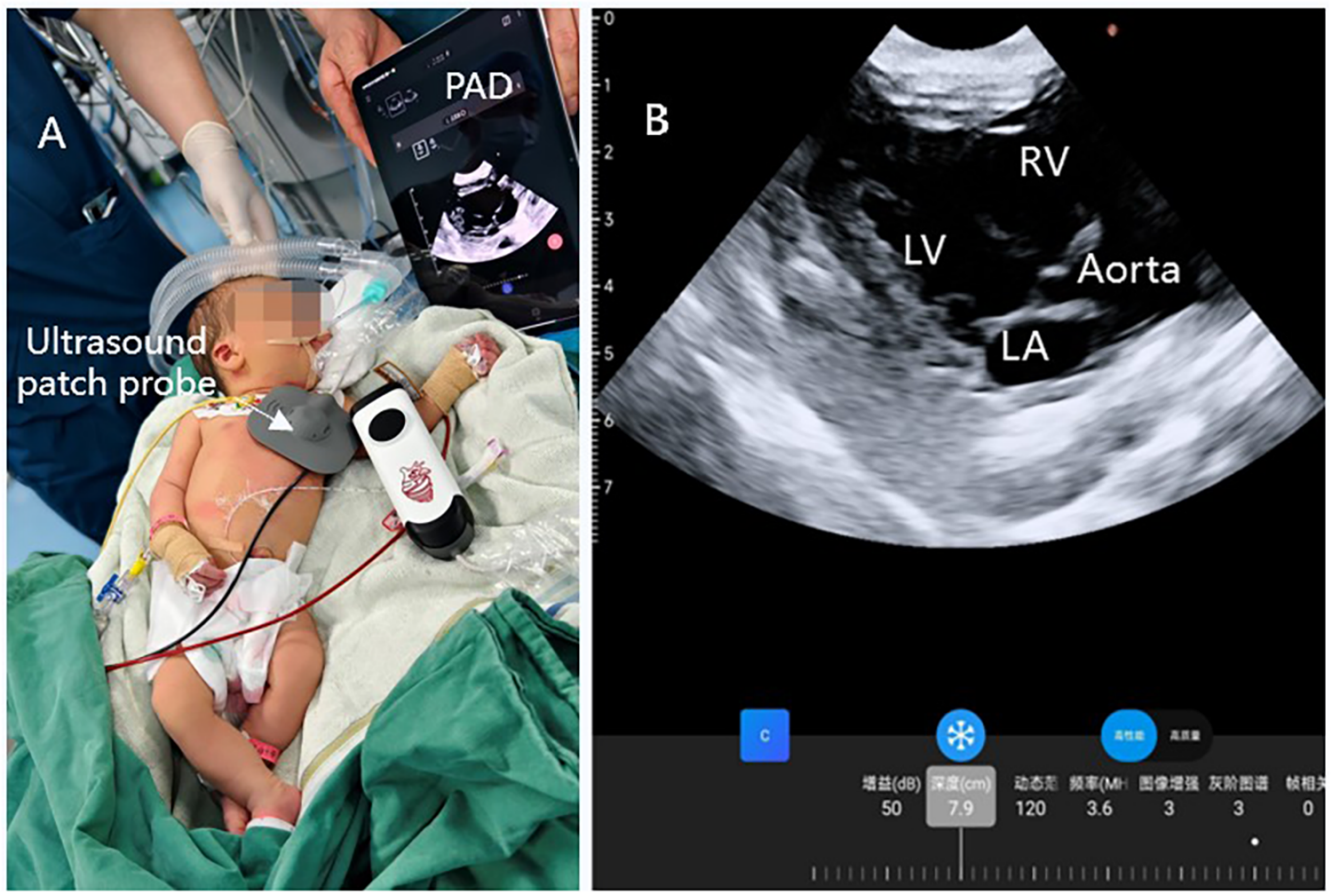
Continuous ultrasound imaging of the heart during operation. **(A)** Wearable ultrasound technology is utilized in anesthesia within clinical settings; **(B)** continuous ultrasound imaging of parasternal long-axis view.

**Figure 4:**
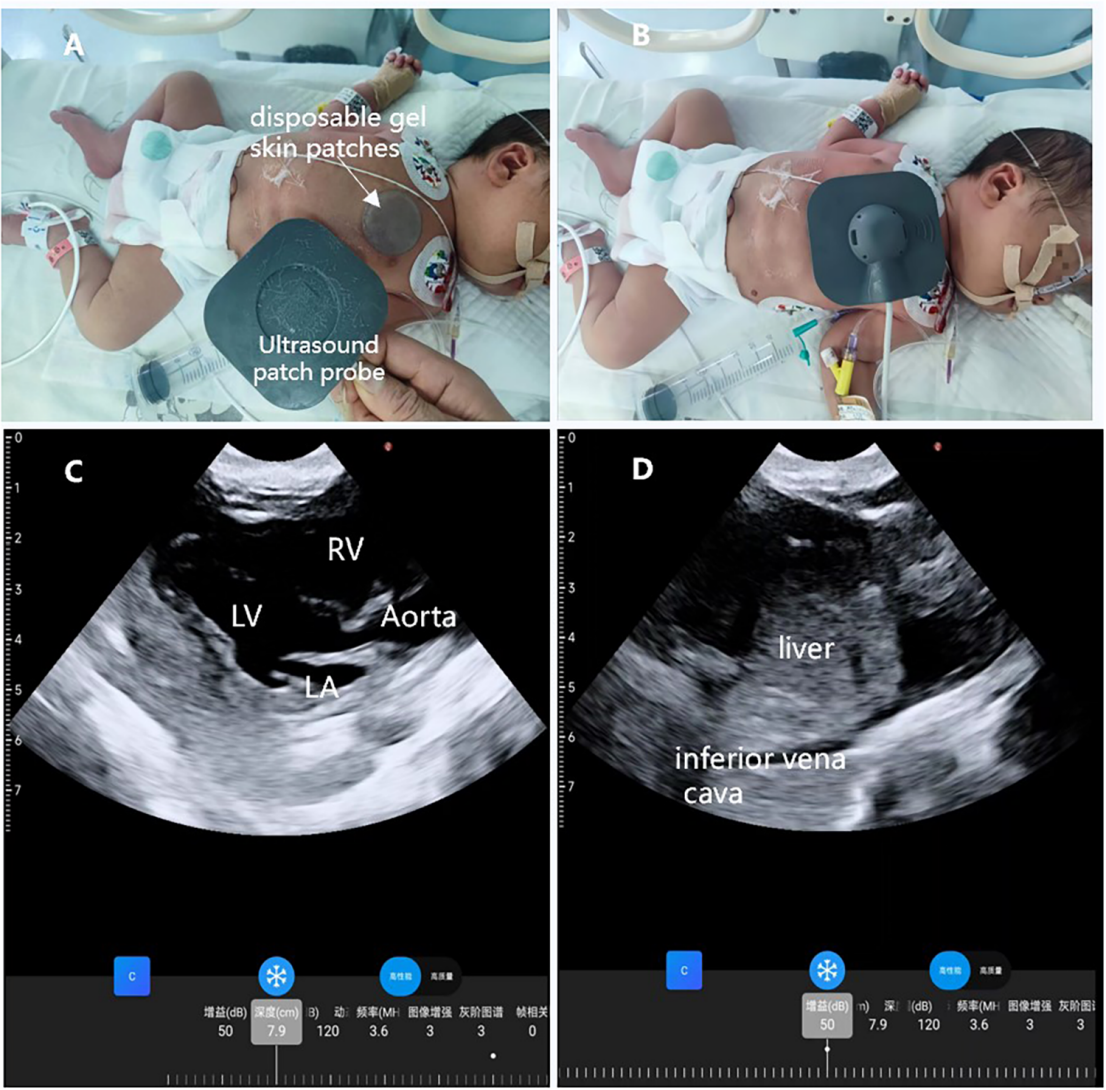
Wearable ultrasound technology is utilized in the NICU. **(A)** A disposable gel skin patches was placed on the neonate's chest for the ultrasound; **(B)** the wearable ultrasound patch was placed on the left anterior chest of the neonate; **(C)** parasternal long-axis view; **(D)** subcostal view of the inferior vena cava.

The neonate exhibited vital signs of HR 145 beats/min, BP 80/60 mmHg, and SPO_2_ 97%. Preoperative ultrasound findings revealed: a left-to-right shunt flow signal with a width of approximately 2.4 mm in the mid-portion of the atrial septum, a shunt signal of approximately 1.6 mm in width between the descending aortic root and the left pulmonary artery, and mild tricuspid valve regurgitation. The results of examination showed that the neonate may have patent foramen ovale (PFO) and patent ductus arteriosus (PDA). In order to ensure the safety of neonatal gastroscopic treatment and anesthesia, we used wearable ultrasound for cardiac monitoring (continuous phased array; model: P2-5-64; 3.6-MHz center frequency, 64 array base, and 0.32-mm array spacing). The probe communicates with the central processor via wireless connection, showed different views of the heart (Figures 1A–D). No PDA shunt was detected during the examination (Figure 2). Following this, the wearable ultrasound probe was secured to the neonate's left anterior chest wall with disposable gel skin patches in order to obtain a parasternal long-axis view. To avoid gastrointestinal bleeding regurgitation and aspiration, we chose endotracheal intubation, utilizing 5 mg of esketamine, 2 mg of rocuronium bromide, and 3 mg of propofol. After induction, The neonate exhibited vital signs of HR 130 beats/min, BP 58/40 mmHg, SPO2 92%, and wearable ultrasound monitoring showed that the left ventricular volume of the heart was slightly deficient, but the contractility was normal (Figures 3A,B; Supplementary Video S1). Based on the echocardiography assessment, we administered 10 ml of crystalloid fluid. The neonate's blood pressure gradually stabilized at 70/40 mmHg. Intraoperative endoscopic observation showed multiple ulcerations in the lower and middle part of the esophagus, and spots of erythema, erosions, and superficial ulcers in the fundus and body of the stomach. After the procedure, the neonate was sent to NICU, where famotidine was given for ulcerations, and wearable ultrasound monitoring was continued to assess myocardial contractility and volume overload (Figures 4A–D; Supplementary Videos S2–S6) to guide the management of fluid replacement and circulation. The neonate's heart rate varied between 130 and 150 beats/min, with SPO2 above 94%(FiO_2_ 40%). The endotracheal tube was removed on the 2nd day. Cardiac ultrasonography on the fifth postoperative day revealed only mild tricuspid regurgitation. The neonate was discharged on the tenth postoperative day.

## Discussion

This case is the first report of the wearable echocardiography used in the perioperative period of neonates. Neonatal anesthesia management is highly challenging due to their unique physiological mechanisms. When hemodynamic instability occurs, limited cardiac reserve increases the risk of postoperative respiratory and cardiac complications compared to other age groups (3).

In this case, the neonate was born with PDA and PFO, which can cause pulmonary vasoconstriction and dilatation of the arterial ducts in the event of hypoxemia or hypercapnia during induction and maintenance of anesthesia; the increase in pulmonary vascular resistance leads to an increase in the right-to-left shunt from the patent foramen ovale and the arterial ducts, which aggravates hypoxemia and hypotension and creates a vicious circle (4), or even cardiac arrest. In addition, due to poor ventricular compliance, excessive fluid infusion may put the neonatal heart at risk of overdistension and heart failure (5). Echocardiography can help us to understand the cardiovascular physiology and anatomy of the neonate, identify congenital cardiac defects, quantify ventricular function and volume to guide clinical decision making during surgery (6, 7). But the traditional probe cannot be fixed on the body surface of the neonate for continuous echocardiographic monitoring. Recent advancements in material design, manufacturing technology, and data processing algorithms have led to the development of wearable ultrasound devices. Xu et al. (8) reported a continuous, real-time wearable ultrasound patch for cardiac function assessment, which was validated in healthy volunteers. In this case, the wearable colour Doppler ultrasound diagnostic device was developed by Sichuan Wismed Medical Technology Co., Ltd. (Chengdu, Sichuan, China), the ultrasound probe adopts a soft structural design, which can naturally fit the skin and maintain a fixed position, and ultrasound images can be wirelessly transmitted to terminal devices such as tablets. Though not specifically designed for neonates, the wearable echocardiography enabled real—time monitoring of the neonatal cardiac status. During the procedure, continuous echocardiography assessed cardiac contractility and volume, guiding fluid management. This indicates that wearable echocardiographic monitoring is effective and feasible for neonates.

## Conclusion

This case confirms that wearable ultrasound with continuous echocardiographic monitoring is practical and feasible for neonatal perioperative circulatory management, offering new ideas for monitoring and managing critical neonates' circulation.

## Data Availability

The original contributions presented in the study are included in the article/Supplementary Material, further inquiries can be directed to the corresponding author.

## Supplementary material

Supplementary Video S1 Intraoperative wearable ultrasound examination: parasternal long-axis view.

Supplementary Video S2 Postoperative wearable ultrasound examination: parasternal long-axis view.

Supplementary Video S3 Postoperative wearable ultrasound examination: parasternal LV short-axis view.

Supplementary Video S4 Postoperative wearable ultrasound examination: parasternal aortic short-axis view.

Supplementary Video S5 Postoperative wearable ultrasound examination: apical 4-chamber view.

Supplementary Video S6 Postoperative wearable ultrasound examination: subcostal view of the inferior vena cava.
